# Impairment in acquisition of conditioned fear in people with depressive symptoms

**DOI:** 10.3389/fpsyg.2024.1384053

**Published:** 2024-05-28

**Authors:** Rui-Han Luo, Feng Su, Xin-Yue Zhao, Tian-Hui Cao, Jing Liao, Yan-Xue Xue, Geng-Di Huang, Jian-Li Yang

**Affiliations:** ^1^Tianjin Medical University General Hospital, Tianjin, China; ^2^Department of Clinical Psychology, Tianjin Medical University General Hospital, Tianjin, China; ^3^Peking-Tsinghua Center for Life Sciences, Academy for Advanced Interdisciplinary Studies, Peking University, Beijing, China; ^4^Tianjin University of Traditional Chinese Medicine, Tianjin, China; ^5^National Institute on Drug Dependence and Beijing Key Laboratory of Drug Dependence, Peking University, Beijing, China; ^6^Department of Addiction Medicine, Shenzhen Clinical Research Center for Mental Disorders, Shenzhen Kangning Hospital, Shenzhen, Guangdong, China

**Keywords:** fear conditioning, associative learning, depression, skin conductance response, hybrid model

## Abstract

**Background:**

Depression is one of the primary global public health issues, and there has been a dramatic increase in depression levels among young people over the past decade. The neuroplasticity theory of depression postulates that a malfunction in neural plasticity, which is responsible for learning, memory, and adaptive behavior, is the primary source of the disorder's clinical manifestations. Nevertheless, the impact of depression symptoms on associative learning remains underexplored.

**Methods:**

We used the differential fear conditioning paradigm to investigate the effects of depressive symptoms on fear acquisition and extinction learning. Skin conductance response (SCR) is an objective evaluation indicator, and ratings of nervousness, likeability, and unconditioned stimuli (US) expectancy are subjective evaluation indicators. In addition, we used associability generated by a computational reinforcement learning model to characterize the skin conductance response.

**Results:**

The findings indicate that individuals with depressive symptoms exhibited significant impairment in fear acquisition learning compared to those without depressive symptoms based on the results of the skin conductance response. Moreover, in the discrimination fear learning task, the skin conductance response was positively correlated with associability, as estimated by the hybrid model in the group without depressive symptoms. Additionally, the likeability rating scores improved post-extinction learning in the group without depressive symptoms, and no such increase was observed in the group with depressive symptoms.

**Conclusion:**

The study highlights that individuals with pronounced depressive symptoms exhibit impaired fear acquisition and extinction learning, suggesting a possible deficit in associative learning. Employing the hybrid model to analyze the learning process offers a deeper insight into the associative learning processes of humans, thus allowing for improved comprehension and treatment of these mental health problems.

## 1 Introduction

Major depression is a mental illness that is characterized by a persistent and significant low mood and is caused by a combination of environmental and hereditary causes, which is widespread, expensive, damaging, and related to heightened risk of suicide. It is a major worldwide public health concern (Marwaha et al., 2023). Rates of depression in youth have sharply increased over the last 10 years, which is concerning because adolescence is a time of rapid changes in social, emotional, and cognitive development, as well as a period of significant life transitions. The risk of recurrence of depression, co-morbidity with other mental illnesses, and more severe and prolonged damages in social, educational, and occupational functioning are the consequences related to depression in young people (Thapar et al., 2022). It has been observed that depression and anxiety are increasingly common among the youth, and they frequently occur together and have similar risk factors (Craske and Waters, 2005). The hypothesis of a shared neurobiological malfunction is supported by the fact that anxiety and depression have many similar symptoms and may respond to comparable therapies. However, the precise neurobiological mechanisms underlying depression and anxiety are not yet fully understood (Nutt et al., 2002). Having more knowledge about depression can help in the development of treatments for other mental health issues.

Fear learning dysfunction is thought to be implicated in the emergence and persistence of an array of psychiatric issues, including not only anxiety disorders (Milad et al., 2014; Otto et al., 2014) and posttraumatic stress disorder (PTSD) (Wicking et al., 2016) but also depression (Sandi and Richter-Levin, 2009). Moreover, studies on extinction learning in depression have yielded conflicting results, one study showed a deficiency (Dibbets et al., 2015), and the other study exhibited enhancement (Kuhn et al., 2014). Therefore, it is believed that maladaptive social anxiety and fear are linked to depression. The ability to recognize and respond to potential danger is essential for survival; however, when this process becomes impaired and when one experiences an abnormal fear response to harmless situations, anxiety disorders may develop. Numerous studies have been conducted for understanding the behavioral, experiential, and neural components of both adaptive and maladaptive fear-learning processes in both animals and humans. Pavlovian fear conditioning is a prevalent model for studying both fear and anxiety, and it continues to influence modern explanations of clinical anxiety issues. Despite its widespread usage in studies of both animals and humans, the neurological basis of fear conditioning is not yet fully comprehended. Investigating the relationship between fear learning and depression can help us gain insights into the associative learning processes of humans, which could lead to improved comprehension and treatment of these mental health issues. Conditioning, extinction, and reinstatement are essential elements of animal adaptation, and they are also closely associated with mental disorders such as PTSD, anxiety, depression, and addictions (Mattera et al., 2020). Fear conditioning and fear extinction learning are fundamental components of models that explain the development of anxiety disorders and the reduction of pathological fear during exposure-based treatments (Shankman and Klein, 2003). Studies conducted on rodent models of learned helplessness have revealed a correlation between impaired fear extinction and heightened depression-like behaviors (Shumake et al., 2005). It was also found that depressed patients had trouble acquiring conditioned fear. Furthermore, following successful fear learning, they displayed impaired extinction of conditioned fear responses (Wurst et al., 2021). Depression increases the risk of enduring persistent fear, which is the characteristic of anxiety disorders, and similarly, persistent fear can contribute to depression.

Fear conditioning is a form of associative learning with adaptive and self-protective functions (Pearce and Bouton, 2001) to predict threat through the association of initially neutral stimuli (conditioned stimuli, CS) with aversive outcomes (unconditioned stimuli, US) (Ojala and Bach, 2020), and the corresponding laboratory model has been used extensively as a classical experimental paradigm to quantitatively measure associative learning (Lonsdorf et al., 2017). Classical formal learning theory, such as the Rescorla–Wagner (R-W) and Pearce–Hall (P-H) models, which explain how the CS and US signals are integrated algebraically to connect cues with aversive events, can provide a good description of computational principles behind direct fear conditioning (LePelley and McLaren, 2004; Lindström et al., 2018). The R-W model is a learning model that is driven by the prediction error, that is, the difference between the expected and actual outcomes of a conditioning trial (Miller et al., 1995; Li and McNally, 2014). In other words, the R-W model suggests that the formation of an association depends on deviations or “errors” between expectations and actuality. However, the P-H model is an associability gated learning model. Organisms learn cue–reinforcer associations by applying a quantity called associability, which is determined by the dynamics of trial-wise prediction errors (Herry and Johansen, 2014). To be more precise, associability is based on prediction errors from prior trials involving the same cue, which indicate the degree to which each cue has been accompanied by a surprise in the past. Learning rate is a constant in the R-W model, but in the P-H model, a cue's associability gates learning rate dynamically, decelerating learning to reliable predictors and accelerating learning to cues that are poor predictors (Li et al., 2011). However, the hybrid model is shown to have better power to comprehend a variety of experimental findings than the previous single model (Le Pelley, 2004).

Recent studies have revealed that people with depression demonstrate improved extinction learning in comparison to healthy controls (Kuhn et al., 2014), as well as enhanced differential acquisition of fear responses toward the CS+ (Nissen et al., 2010). Despite contrary reports (Otto et al., 2014; Waters et al., 2014; Wurst et al., 2021) or null findings (Dibbets et al., 2015), most studies have been conducted with relatively small sample sizes, making it necessary to conduct more research to gain a better insight into the effects of depression on fear acquisition and extinction learning. The neuroplasticity hypothesis of depression postulates that a malfunction in neural plasticity, which is responsible for learning, memory, and adaptive behavior, is the primary source of the disorder's clinical manifestations (Spedding et al., 2003; Normann et al., 2007; Kuhn et al., 2014). Due to the attentional and memory deficits associated with mood disorders, we predicted that the people with depressive symptoms would show poor acquisition of the conditioned fear response than those without depressive symptoms. This is because differential conditioning necessitates the learning and subsequent discrimination of the meaning of both a CS+ and CS–.

## 2 Materials and methods

### 2.1 Subjects

In total, 53 healthy college students aged between 18 and 30 years participated in this 2-day study. On the 1^st^ day of the experiment, four subjects showed flat SCRs, which were deemed as a failure to show detectable or reliable SCR. Therefore, these four subjects were excluded. On the 2^nd^ day of the experiment, three subjects were unable to participate in the experiment due to personal reasons, and for two other subjects, SCR and subjective evaluation data were not collected due to equipment failure. Therefore, finally, 49 subjects participated on the 1^st^ day of the experiment and 44 on the 2^nd^ day of the experiment. All participants were right-handed with self-reported normal vision or corrected vision. Written informed consent was obtained from all participants. The experimental procedure was approved by the Ethics Committee of the Tianjin Medical University and Peking University.

### 2.2 Assessment of psychiatric symptoms

All subjects were required to complete five self-report questionnaires, which were brief, commonly used, and psychometrically sound, before starting the task on the 1^st^ day.

The Beck Depression Inventory (BDI) consists of 13 items, and each item is rated on a 4-point scale from 0 to 3. The BDI is administered to measure depressive symptoms at this moment. A total score is used to distinguish the presence and severity of depressive symptoms: 0–4 represents (basically) no depressive symptoms, 5–7 is mild, 8–15 is moderate, and 16 or more is severe (Beck et al., 1961; Metcalfe and Goldman, 1965; Aalto et al., 2012). The State-Trait Anxiety Inventory (STAI) consists of two subscales: the State Anxiety Inventory (S-AI) and the Trait Anxiety Inventory (T-AI), each with 20 items, which can assess current (state anxiety) and persistent (trait anxiety) anxiety symptoms, respectively. Each item is scored on a scale of 1–4. Cumulative scores for the S-AI and T-AI subscales are calculated separately, with a minimum of 20 and a maximum of 80, and then summed to calculate the total score. Based on the age of the subjects included in this study, the thresholds were used as follows: male subjects with state anxiety scores over 53 and trait anxiety scores over 56 and female subjects with state anxiety scores over 55 and trait anxiety scores over 57 were considered abnormal. Higher scores indicate higher levels of anxiety (Spielberger et al., 1971; Spielberger, 1983).

The Childhood Trauma Questionnaire (CTQ) is used to assess childhood adverse experiences before the age of 16 years. The CTQ has a total of 28 items. Each item is graded on a 5-point scale. The CTQ can be divided into five subscales, each of which is scored between 5 and 25 points. When emotional abuse ≥ 13, sexual abuse ≥ 8, physical abuse ≥ 10, emotional neglect ≥ 15, and physical neglect ≥ 10, any subscale score meeting the above criteria is considered as accompanied by childhood trauma (Bernstein et al., 2003).

The Pittsburgh Sleep Quality Index (PSQI) evaluates the sleep quality of the subjects in the last month. The items consist of seven components, and each component is scored on a scale from 0 to 3. The total score of the PSQI is the sum of the scores of the seven components, ranging from 0 to 21. Higher scores indicate poorer sleep quality, using seven points as the threshold (Buysse et al., 1989).

The Zhang Ming Yuan Life Event Scale (LES) has a total of 65 items, covering a variety of common life events. According to the age of the subjects, the corresponding value for the life event unit (LEU) was taken. The LEU value of each event in the past year was added to get the total LEU value.

### 2.3 Conditioning paradigm

On the 1^st^ day, all participants underwent a differential fear conditioning paradigm. Two geometric shapes (circle and triangle) as non-conditioned (CS–) and conditioned (CS+) stimuli were presented on a gray background, with black color, the same size, and mean luminance. The assignment of shapes to the CS type was balanced across subjects. The order of the trial was generated pseudo-randomly with two constraints. The first trial was always CS+US, and no more than two consecutive trials of the same kind were observed. The US usually was an aversive outcome. Here, a mild electric shock was delivered to the participant's right forearm via the BIOPAC stimulator module STM200-1 (BIOPAC Systems Inc., Goleta, California, USA), which served as the US. The intensity of the shocks was customized for everyone to a level described as “aversive but not painful.” The experiment was performed by administering a constant shock intensity throughout. The conditioned stimulus (CS+) was presented for 4 s and was paired with an electric shock of 500-ms duration and 50 Hz current pulse, with the application of partial reinforcement (50%). The conditioned stimulus was never paired with an electric shock (CS–). The conditioning phase consisted of 24 CS+ trials, 12 of which were reinforced with the electric shock, and 12 CS– trials. Partial reinforcement was used to delay acquisition and to evaluate the response to the CS+ without interference from the shock or habituation to the shock. During an inter-trial interval randomly determined to last 7–11 s, a fixation cross was shown at the center of the gray background. Subjects were instructed to focus on the geometric shapes presented on the computer screen and to discern the correlation between the stimuli and shocks. Before the conditioning phase, a habituation phase was conducted without electrical shock, wherein the CS+ and CS– were each displayed four times randomly, amounting to eight trials, to accustom the physiological response to the novel stimulus (Boucsein et al., 2012).

After 24 h, subjects participated in extinction training. The extinction phase consisted of 24 trials: 12 CS+ trials without the US pairing and 12 CS– trials. The trials remained in a pseudo-random order with no more than two sequential presentations of either the CS+ or CS–. During the extinction phase, no electric shocks appeared throughout the process. Subjects were given no instructions to changes in the previous CS-US contingency. Each stimulus was also displayed for 4 s, with the same inter-trial interval as applied in the conditioning phase.

**Figure 1:**
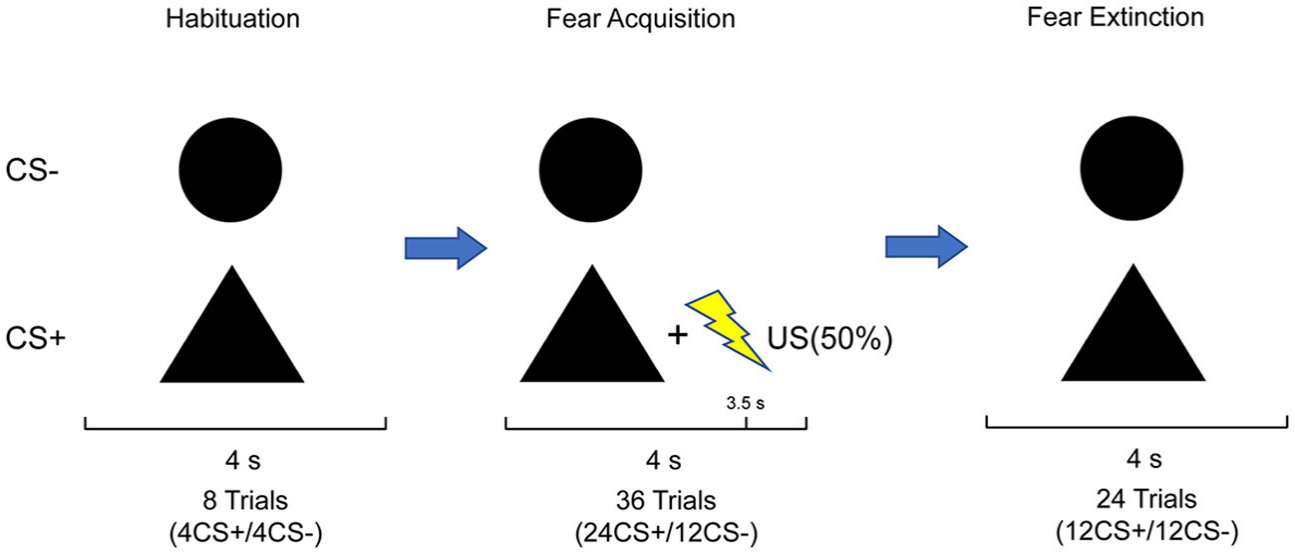
Stimulus set and experimental design: the paradigm comprised three phases. The habituation phase consisted of four CS+ and four CS–; no US was presented. The acquisition phase consisted of 24 CS+ and 12 CS–. The CS+ co-terminated with the US in 50% of trials, whereas the CS– was never paired with the US. The extinction phase consisted of 12 CS+ and 12 CS–, in which CS+ and CS– were never paired with US. Subjects were not informed about the CS–US contingencies.

In addition, subjects were required to rate the nervousness, likeability, and US expectancy of each CS following each experimental phase. Nervousness and likeability were rated on a 7-point-Likert scale, ranging from “very relaxed” (1) to “very nervous” (7) and “very dislike” (1) to “very like” (7), respectively. US expectancy was indicated as the probability of a shock following each stimulus in percent on a scale from 1 to 100 in 10% steps. The whole task procedure (Figure 1) was run by The Psychtoolbox Version 3 of MATLAB R2022b.

### 2.4 Physiological assessment

The skin conductance response (SCR) is one of the most common physiological indicators to assess human threat learning (Lonsdorf et al., 2017), which is often measured to make inferences about mental processes such as emotional arousal and threat prediction (Boucsein et al., 2012). The SCR was assessed using two Ag–AgCl electrodes, filled with standard NaCl electrolyte gel, which were attached to the index and middle fingers of the left hand, between the first and second phalanges. Signals were amplified and recorded with a BIOPAC MP 150 (BIOPAC Systems Inc., Goleta, California, USA) skin conductance module connected to an HP computer. Data were continuously recorded at a rate of 2,000 Hz, and SCR waveforms were analyzed offline using AcqKnowledge 5.0 software (BIOPAC Systems Inc., Goleta, California, USA) with a notch filter of 50 Hz.

The SCR level for each trial was determined by measuring the base-to-peak difference for the waveform (in micro siemens, μs) in the 0.5 to 9.5 s window following stimulus onset. A minimal response standard of 0.02 μs was established; if the SCR response was lower than this, it was recorded as 0. The raw SCR scores were square root-transformed to normalized distributions (Olsson et al., 2007; Schiller et al., 2008, 2010, 2012, 2013). Trials affected by body movements, coughing, sneezing, or other involuntary behaviors were removed based on the abnormal waveform.

### 2.5 Data analysis

The descriptive presentation of the data included mean values and standard deviations. We used IBM SPSS Statistics 26 to conduct an independent sample *t*-test and chi-square test on demographic characteristics and the scores of the five scales for group comparisons. The researchers conducting the data analysis were blinded to the group assignments to minimize potential bias and confounding factors. A 2 × 2 × 2 repeated measures analysis of variance (ANOVAs) was conducted to examine the main effects and interactions of subjective evaluations, with phase (acquisition and extinction) and stimulus (CS+ and CS-) as within-subject factors and group (BDI > 4, BDI ≤ 4) as between-subject factor. ANOVAs were followed by simple effect analysis where applicable and necessary. For the SCR, a paired-sample *t*-test was used. The level of significance was set at *p* < 0.05.

To further explore the differences between the two groups, we chose the hybrid model to fit the SCR data of each subject (i.e., using a separate set of free parameters for each subject to achieve the optimal fitting). The hybrid model was as follows:


$$\begin{array}{l} \;\delta_{n}\;=\;r_{n}-V_{n}\left(x_{n}\right)\\ V_{n+1}\left(x_{n}\right)\;=\;V_{n}\left(x_{n}\right)+k\alpha_{n}\left(x_{n}\right)\delta_{n}\\ \alpha_{n+1}\left(x_{n}\right)\;=\;\eta \left|\delta_{n}\right|+\left(1-\eta \right)\alpha_{n}\left(x_{n}\right) \end{array}$$

In the model, x_n_ was defined as a conditioned stimulus on trial n (CS+ or CS–) and r_n_ as the US (1 for US; 0 for no US). V_n_(x_n_) indicated value predictions (i.e., shock) for each stimulus type and trial. The prediction error δ_n_ referred to the difference between the actual US (i.e., r_n_) and the expected US [i.e., V_n_(x_n_)] on trial n. The α_n_(x_n_) meant the associability estimated by the hybrid model for each stimulus type and trial. Additionally, η represented the weight assigned to the latest PE, and κ was the normalization factor. The PE weights (η) were constrained to the range (0, 1) with a β (1.2, 1.2) prior distribution slightly favoring values in the middle of the range; normalization factor κ was constrained to be positive values with a γ (1.2, 1) prior distribution (Raio et al., 2017; Dunsmoor et al., 2019).

In the hybrid model, the initial values of α_0_ and V_0_ were set as 0.5 for all kinds of stimuli. Moreover, we calculated the average of the SCR and associability estimated by the computational model for each trial within all subjects in each group. Then, we calculated the Pearson correlation and made linear regressions from the associability to the SCRs and achieved the best fit through the least squares. All US trials were omitted from the regression onto the SCRs to avoid possible contamination of the predictive response by shock-related responses, but they were included in the computation of associability.

## 3 Results

### 3.1 Subject characteristics

Subjects were categorized into two groups according to BDI scores: the group with depressive symptoms (total score > 4) and the group without depressive symptoms (total score ≤ 4). Groups did not differ in demographic characteristics, such as age [t_(47)_ = 0.560, *p* = 0.578], sex (χ^2^ = 0.420, *p* = 0.517), and educational level [t_(47)_ = 0.470, *p* = 0.641]. Table 1 shows that the group with depressive symptoms had higher scores in S-AI, T-AI, PSQI, and CTQ than the group without depressive symptoms, although these scores were within the normal range.

**Table 1:** The demographic data of individuals with and without self-rated depressive symptoms.

	**BDI ≤ 4 (*n* = 29)**	**BDI > 4 (*n* = 20)**	**χ2/t (*p*-value)**
**Demographic variables**			
Age	24.45 (2.080)	24.10 (2.222)	0.560 (0.578)
Range	21–30	20–28	
Sex (M:F)	9:20	8:12	0.420 (0.517)
Education, years	18.38 (1.916)	18.10 (2.222)	0.470 (0.641)
**Scales rating**			
BDI	1.34 (1.396)	9.85 (4.308)	−8.527 (0.000)
S-AI	31.97 (6.179)	38.95 (7.149)	−3.647 (0.001)
T-AI	35.10 (6.810)	45.30 (7.197)	−5.034 (0.000)
PSQI	3.90 (1.739)	5.55 (1.468)	−3.479 (0.001)
CTQ	32.46 (6.269)	37.82 (8.988)	−2.308 (0.026)
LES	70.96 (50.149)	104.35 (90.227)	−1.392 (0.178)

Subjects were grouped according to their BDI scores. The value represents mean ± SD. The *p*-value for the statistical tests: chi-square test for sex distribution and Student two-sample *t*-test (unequal variance assumed) for the questionnaire variables.

### 3.2 Subjective evaluations

**Figure 2:**
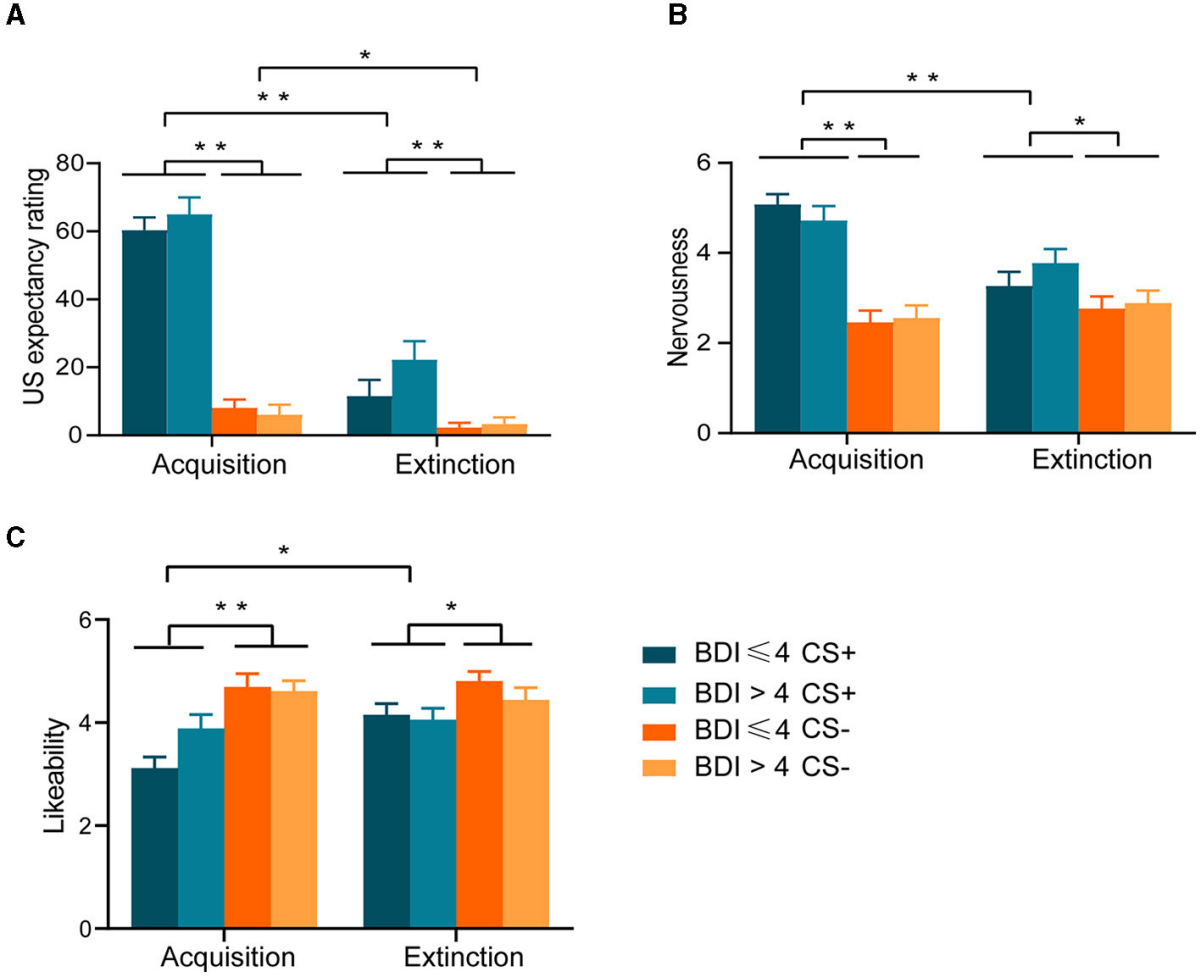
Results of 2 × 2 × 2 repeated-measures ANOVA for subjective evaluations, **(A)** Unconditioned stimulus (US) expectancy ratings. **(B)** Nervousness ratings. **(C)** Likeability ratings *p < 0.05; **p < 0.001.

For US expectancy, the ANOVA yielded a significant Phase × Stimulus interaction [*F*_(1, 42)_ = 100.254, *p* < 0.001]. Follow-up simple effect analysis further revealed higher levels of US expectancy to the CS+ relative to CS– after the acquisition (*p* < 0.001) and extinction (*p* < 0.001), and a higher level of US expectancy to the CS+ was reported after the acquisition than extinction (*p* < 0.001), the same being true for CS– (*p* = 0.037). In addition, the main effect of phase was significant [*F*_(1, 42)_ = 106.498, *p* < 0.001]. Finally, the main effect of the stimulus was also significant [*F*_(1, 42)_ = 180.450, *p* < 0.001]. Between-group effects were not significant [*F*_(1, 42)_ = 1.183, *p* = 0.283] (Figure 2A).

For nervousness ratings, the ANOVA yielded a significant Phase × Stimulus interaction [*F*_(1, 42)_ = 40.100, *p* < 0.001]. Follow-up simple effect analysis further revealed higher levels of nervousness to the CS+ relative to CS– after the acquisition (*p* < 0.001) and extinction (*p* = 0.003), and a higher level of nervousness to the CS+ was reported after the acquisition than extinction (*p* < 0.001), but not for CS-. In addition, the main effect of phase was significant [*F*_(1, 42)_ = 9.018, *p* = 0.004]. Finally, the main effect of the stimulus was also significant [*F*_(1, 42)_ = 51.613, *p* < 0.001]. Between-group effects were not significant [*F*_(1, 42)_ = 0.118, *p* = 0.732] (Figure 2B).

For likeability ratings, the ANOVA yielded a significant Phase × Stimulus interaction [*F*_(1, 42)_ = 6.791, *p* = 0.013] and a significant Phase × Group interaction [*F*_(1, 42)_ = 6.966, *p* = 0.012]. Follow-up simple effect analysis further revealed lower levels of likeability to the CS+ relative to CS– after the acquisition (*p* < 0.001) and extinction (*p* = 0.009), and a lower level of likeability to the CS+ was reported after the acquisition than extinction (*p* = 0.001), but not for CS-. Moreover, likeability increased in the group without depressive symptoms from acquisition to extinction (*p* < 0.001), while not in the group with depressive symptoms. In addition, the main effect of phase was significant [*F*_(1, 42)_ = 6.966, *p* = 0.012]. Finally, the main effect of the stimulus was also significant [*F*_(1, 42)_ = 15.971, *p* < 0.001]. Between-group effects were not significant [*F*_(1, 42)_ = 0.093, *p* = 0.761] (Figure 2C).

### 3.3 Skin conductance response

**Figure 3:**
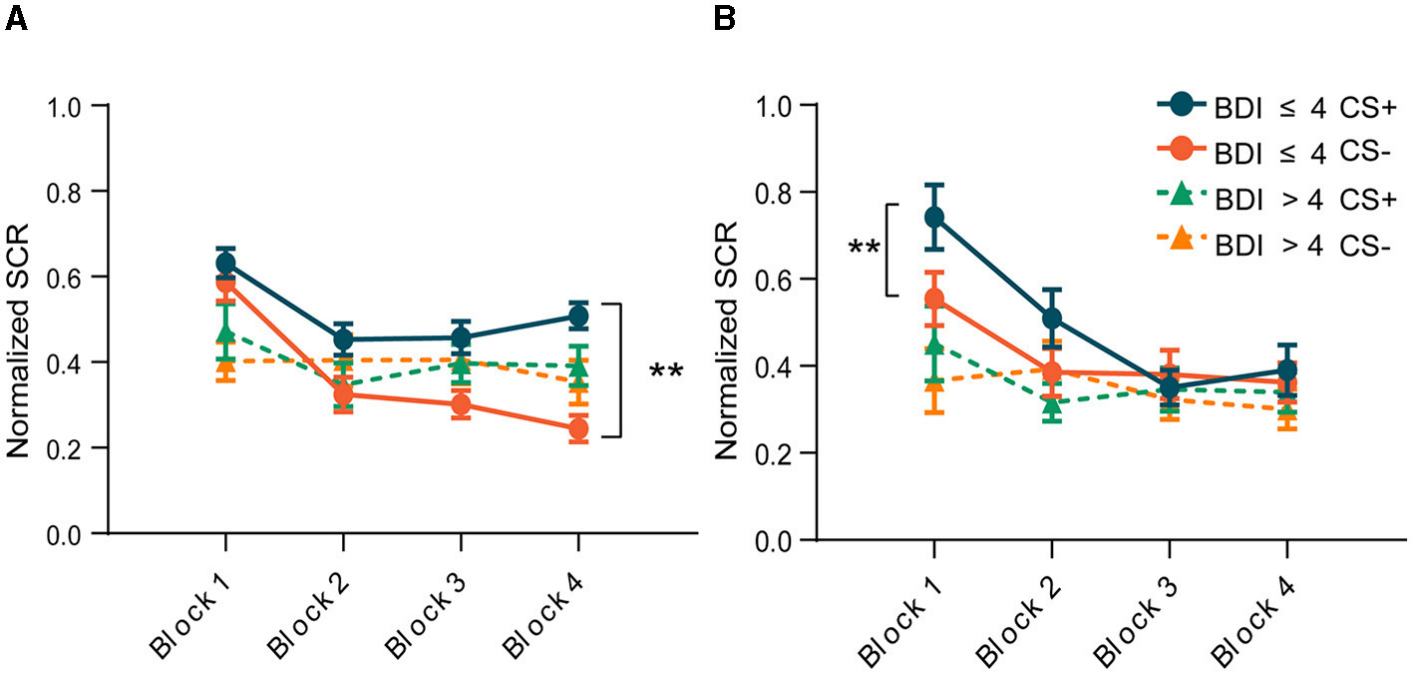
Changes in skin conductance responses throughout the experiment within the two groups. **(A)** Acquisition phase. **(B)** Extinction phase. **p < 0.001.

To avoid the confounding effect of the US on the SCR, trials paired with electric shock were excluded from the SCR analysis. In addition, to capture the time course of differential fear conditioning, we divided each phase into four blocks, three CS+ and three CS– in each block, and calculated the average of the SCR in each block. Figure 3 depicts the mean SCR for each group across each phase. The criterion of the SCR for successful acquisition was defined as the conditioned threat responses (CRs) in the last block being >0.05. Conditioned threat responses (CRs) were calculated by subtracting the mean SCR to CS- from that of CS+ (CS+ minus CS–). The result of the paired-sample *t*-test indicated that the group without depressive symptoms exhibited heightened SCRs to the CS+ vs. the CS– in the last block during fear conditioning [*t*_(28)_ = −7.733, *p* < 0.001, Figure 3A], and the conditioned SCR was significantly higher than 0.05 [*t*_(28)_ = 6.268, *p* < 0.001], indicating successful acquisition of conditioned fear. The primary index of fear recall (Day 2) was the mean SCR to the early CS+ vs. CS– trials. Our results showed that in the first two blocks of the extinction phase, the differential responses reappeared in the group without depressive symptoms [*t*_(25)_ = −4.273, *p* < 0.001, *t*_(25)_ = −2.874, *p* = 0.008, Figure 3B]. After several unreinforced trials, subjects began to extinguish, manifested by the disappearance of the difference between CS+ and CS–. However, subjects in the group with depressive symptoms showed similar responses to CS+ and CS– throughout the acquisition phase [*t*_(19)_ = −0.376, *p* = 0.711, Figure 3A] and the extinction phase [*t*_(17)_ = −0.651, *p* = 0.524, Figure 3B]. This result suggests that subjects with depressive symptoms did not establish a conditioned fear, and thus there was no extinction.

### 3.4 Computational model fitting

**Figure 4:**
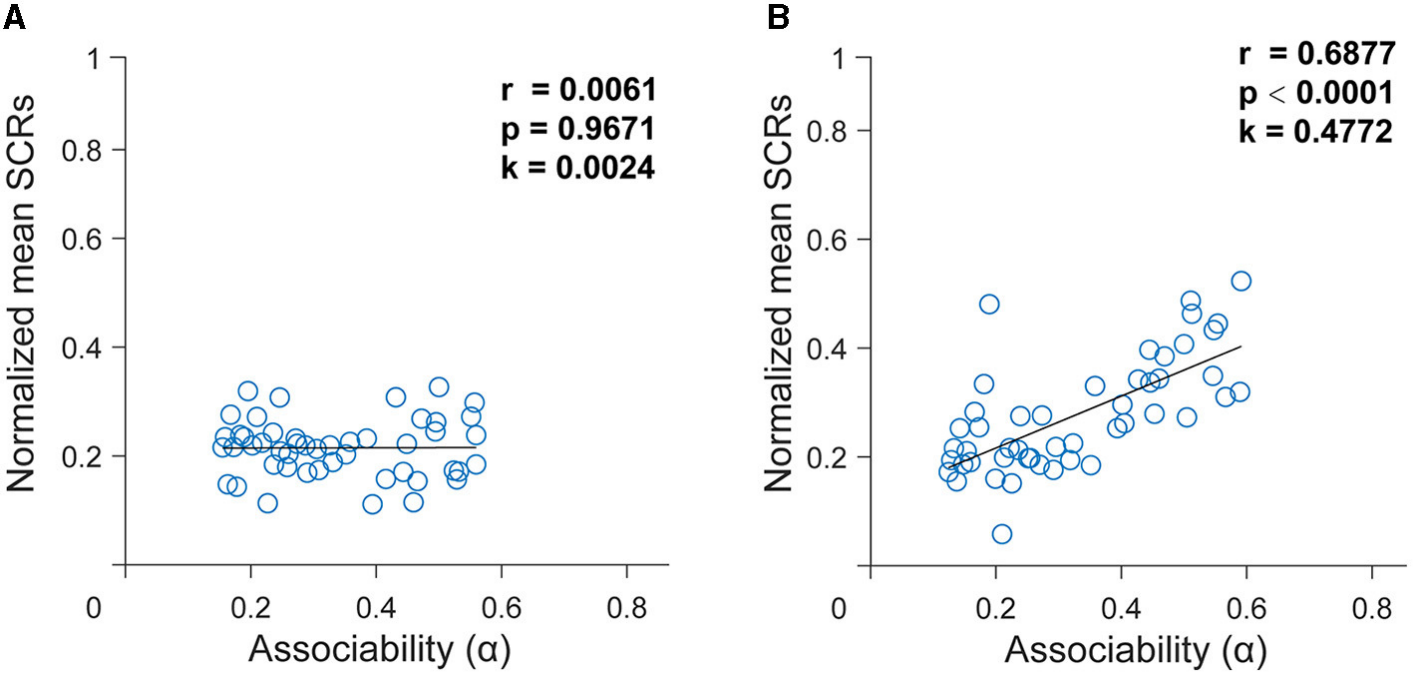
Dynamics of associability (α) generated from the best-fitting hybrid model. **(A)** In the group with depressive symptoms. **(B)** In the group without depressive symptoms. Relationship between associability (α) and skin conductance responses (SCRs) in two groups using the best fitting hybrid model. Data represent average SCRs for each trial averaged across subjects. The black curve is the best-fitting line using least squares.

To test whether the SCR data were consistent with the hypothesized learning mechanisms, we fitted SCR data using the hybrid model. After using the best setting of the free parameters, when applied to the trial-by-trial time series of the SCR, the hybrid model defined the associability or dynamic learning rates that might be reflected in the SCR. To further illustrate the extent to which associability explains the SCR, then we performed linear regressions from the associability to the SCR. There was no correlation between the associability derived from the group with depressive symptoms and the SCR [*r* = 0.0061, *p* = 0.9671, k = 0.0024, Figure 4A]. By contrast, applying the same analysis, results revealed a significant correlation between associability and the SCR in the group without depressive symptoms [*r* = 0.6877, *p* < 0.0001, k = 0.4772, Figure 4B]. This indicates that the associative learning model fitted better in the group without depressive symptoms and subjects in the group with depressive symptoms had impaired associative learning.

## 4 Discussion

The current study explored basic forms of learning to further test the neuroplasticity hypothesis of depressive symptoms in associative learning. The strengths of the study include a well-defined sample of participants with depressive symptoms and participants without depressive symptoms matched according to sex, age, and educational level. Based on the results of the skin conductance responses, we found that the subjects with depressive symptoms had deficits in fear acquisition. Furthermore, we used associability as estimated by a hybrid model to investigate the effect of depressive symptoms on the associative learning process. We found that skin conductance responses of subjects without depressive symptoms, who successfully acquired differential conditioned fear, were positively correlated with associability, as estimated by a hybrid model.

During a typical differential fear conditioning procedure, subjects learn to form an association between CS+ and US. The CS+ becomes a predictor of the US and will itself evoke the associated fear, known as the conditioned response (CR), which consists of motor, neuroendocrine, autonomic, and other components (Krabbe et al., 2018). Differential skin conductance and subjective responses to the CS+ than the CS– reflect that the subject has successfully acquired the conditioned fear responses (Schiller et al., 2010; Rainer et al., 2020). In the present study, the results of the SCR showed that the subjects with depressive symptoms did not successfully acquire conditioned fear, although subjective evaluations showed that they exhibited higher US expectancy, nervousness, and likeability ratings to CS+. These findings were anticipated because the dissociation between the subjective evaluations and SCR results was common in previous studies (Britton et al., 2013; Shechner et al., 2015, 2018; McLaughlin et al., 2016). McLaughlin et al. also measured the SCR continuously and collected subjective evaluations after each phase. They found disturbances in the acquisition of conditioned fear in maltreated children based on the SCR results. Maltreated children exhibited blunted SCR to the CS+ and failed to exhibit differential SCR to the CS+ vs. CS- during early conditioning relative to healthy controls. However, all subjects reported greater fear of CS+ than CS– after conditioning, regardless of maltreatment (McLaughlin et al., 2016). Unconditional stimulation anticipation evaluations and contingency awareness tests suggested high and similar cognitive representations of the association between the CS and the US in depression patients and healthy controls (Blair et al., 2001; Phelps and LeDoux, 2005; Vansteenwegen et al., 2008). In the present study, both groups showed no difference in the SCR between CS+ and CS– at the end of the extinction phase. However, the likeability ratings of CS+ did not increase in the group with depressive symptoms after extinction. These results were consistent with those of Shechner et al. (2018), who indicated successful extinction for the sample, as indexed by the SCR. However, subjective evaluations showed that the difference between CS+ and CS– still exists. Subjective evaluations may represent a more cognitive expression of threat anticipation, reflecting declarative knowledge of stimulus contingencies. The SCR can track emotional arousal change resulting from exposure to the stimulus, even upon subliminal conditions (Raio et al., 2012).

Though anxiety and depression frequently co-occur, only a few studies have been conducted on fear conditioning in depression, with inconclusive results. Our results were similar to those of Otto et al. (2014) and Wurst et al. (2021), who found significantly attenuated differential fear conditioning among depression patients. In other words, compared to the healthy individuals, patient samples were conditioned less well. Additionally, one study on fear conditioning in offspring susceptible to affective disorders due to maternal psychopathology found that the offspring of depressed mothers showed diminished responses to aversive cues during the acquisition phase (Waters et al., 2014). However, Nissen et al. (2010) conducted a classical differential fear conditioning paradigm to explore the fear acquisition of major depressive disorder patients and healthy controls, wherein a mild electric shock was used as the unconditioned stimulus (US) and four geometric shapes served as the conditioned stimulus (CS). However, the reduced differential skin conductance responses in the group with depressive symptoms were not in line with the results of that study, which showed that MDD patients had a greater skin conductance response (SCR) to the CS+ than to the CS–, while the control participants did not demonstrate differential SCRs to the CSs. Although the study by Nissen et al. showed that patients with major depressive disorder successfully acquired conditioned fear, the anxiety levels of the MDD patients were not taken into account in that study. Given that the MDD patients are often accompanied by anxiety symptoms, it is possible that anxiety contributed to the acquisition of conditioned fear in the MDD patients. A meta-analysis of fear conditioning in anxious and non-anxious adolescents showed that successful acquisition of conditioned fear occurred in both groups, demonstrating comparable differential learning, but anxious individuals had higher fear responses to both CS+ and CS– than non-anxious individuals (Dvir et al., 2019). These results are consistent with those of two previous meta-analyses of fear acquisition in anxious and non-anxious adults (Lissek et al., 2005; Duits et al., 2015), suggesting some combination of stronger excitatory processes to the CS+ and weaker inhibitory processes to the CS– in anxiety. In the present study, although there were differences in anxiety levels between the two groups, they were within the normal range. Thus, our results better eliminated the influence of anxiety symptoms and strongly suggested that impaired conditioned fear acquisition was due to depressive symptoms.

Previous results from skin conductance measurement revealed a lessened fear acquisition among the patient samples, particularly in individuals with depression and posttraumatic stress disorder, indicating an attenuated fear conditioning (Otto et al., 2014). Studies have indicated that individuals suffering from depression, particularly endogenous depression, tend to have lower levels of skin conductance and a higher percentage of SCR non-responders to unpleasant auditory stimuli than healthy persons (Lader and Wing, 1964; Mirkin and Coppen, 1980). The results of this study suggest that people with depression may not be as affected by external stimulation (Mirkin and Coppen, 1980), likely because of the wide-reaching and persistent nature of depressive disorders weakening the human defensive system, thereby decreasing psychophysiological reactivity to emotional stimuli (McTeague et al., 2012). Previous research has indicated that depression is associated with a reduction in fear-potentiated startle and pleasure-inhibited startle, leading to a generally flattened affect–startle pattern (Vaidyanathan et al., 2012). This could explain why the depression group in the current study exhibited similar skin conductance responses to CS+ and CS–. Consequently, these studies sought to investigate whether a weakened psychophysiological response is a risk marker that develops before the onset of depression in those predisposed to the disorder.

Rescorla–Wagner's empirically well-supported notion of error-driven value update was incorporated into the Pearce–Hall associability model, resulting in the hybrid model (Li et al., 2011). These models are usually fitted to the trial-by-trial skin conductance responses in threat learning (Pearce and Hall, 1980; LePelley and McLaren, 2004). We found that the skin conductance responses of subjects without depressive symptoms who successfully acquired differential conditioned fear were positively correlated with associability, as estimated by a hybrid model. It has been proposed that complex fear-learning procedures might be more effective than the traditional differential fear-conditioning paradigm in detecting individual differences related to susceptibility to anxiety disorders. This is based on the idea that those at risk of developing an anxiety disorder may have an abnormal fear response to a real-life conditioning event (Arnaudova et al., 2013). Consequently, further exploration of complex fear learning may be critical for gaining insight into the general principles of associative learning in humans, as real-world Pavlovian associations are likely more intricate than those explored in laboratory experiments.

## 5 Conclusion

Our study revealed that those with self-reported depressive symptoms may have impairments in associative learning. The effect of depressive symptoms on fear conditioning is distinct from that of anxiety symptoms. Different outcomes of differential fear conditioning may aid in distinguishing depression from anxiety. Understanding the physiological differences that may be the source of symptomatology is vital for understanding the varied pathophysiology of these disorders.

## Data availability statement

The raw data supporting the conclusions of this article will be made available by the authors, without undue reservation.

## Ethics statement

The studies involving humans were approved by the Ethics Committee of the Tianjin Medical University. The studies were conducted in accordance with the local legislation and institutional requirements. The participants provided their written informed consent to participate in this study.

## Author contributions

R-HL: Data curation, Formal analysis, Writing—original draft, Writing—review & editing, Investigation. FS: Formal analysis, Software, Writing—review & editing, Data curation. X-YZ: Writing—review & editing, Investigation. T-HC: Writing—review & editing, Investigation. JL: Writing—review & editing, Investigation. Y-XX: Funding acquisition, Supervision, Writing—review & editing, Conceptualization, Project administration. G-DH: Data curation, Software, Writing—review & editing, Project administration. J-LY: Writing—review & editing, Project administration.
